# Frequent accelerated idioventricular rhythm in an otherwise healthy child: a case report and review of literature

**DOI:** 10.1186/s12872-023-03074-5

**Published:** 2023-01-20

**Authors:** Daria Ljubas Perčić, Nikola Krmek, Ivica Benko, Hrvoje Kniewald, Suzana Bitanga, Matej Katavić, Marko Perčić

**Affiliations:** 1 Department of Neonatology, University Hospital “Sveti Duh”, Zagreb, Croatia; 2 Department of Pediatrics, University Hospital Center “Sestre Milosrdnice”, Zagreb, Croatia; 3 Department of Cardiology, University Hospital “Sveti Duh”, Zagreb, Croatia

**Keywords:** Accelerated idioventricular rhythm (AIVR), Frequent AIVR, Pediatric, Radiofrequent ablation, Case report

## Abstract

**Background:**

Accelerated idioventricular rhythm (AIVR) is a wide QRS complex dysrhythmia that, as far as pediatric population is concerned, occurs mostly in children with underlying systemic or heart disease. Its clinical course is thought to be typically benign in otherwise healthy children and treatment to be completely needless. Existing guidelines/recommendations are based entirely on cases that had low daily burden of AIVR, and those referring to treatment itself are very unspecific. Pharmacologic therapy has been mostly unsuccessful and catheter ablation as a way of treatment has been only sporadically reported. This article is a case report with a literature review that aims to practically separate the age groups into newborn and older children and to emphasize the different clinical outcomes of children with occasional and frequent AIVR. There are only a few cases so far describing undesirable outcomes of this condition, and most of these patients had high daily burden of AIVR. To be more specific, among 38 healthy children older than 1 year reported in total, 6 had undesirable outcomes, short-term in terms of developing malignant arrhythmia or long-term in terms of developing cardiomyopathy/heart failure.

**Case presentation:**

An 11-year-old boy had been referred to our center for a workup of incidentally discovered wide-complex arrhythmia. He was asymptomatic, with no underlying cardiac or systemic diseases. Continuous heart rate monitoring detected AIVR during most time of monitoring. In 24-h Holter-ECG, wide QRS complexes accounted for 73%. With parental consent, we conducted an electrophysiological study accompanied by radiofrequent ablation of ectopic focus, which lead to an instantaneous sinus rhythm that continued during the entire follow-up.

**Conclusion:**

AIVR is a rare dysrhythmia in the pediatric population, typically considered benign. Nevertheless, more than a few cases evidence its harmful potential, short-term in terms of developing malignant arrhythmia or long-term in terms of developing cardiomyopathy. Gathering more knowledge and experience along with conducting further studies is essential for the enhancement of understanding this condition, and selecting potentially vulnerable patients as well as their treatment.

## Introduction

Accelerated idioventricular rhythm (AIVR) is a wide QRS complex arrhythmia that is consisted of at least three consecutive ventricular complexes which are mostly monomorphic [1]. It is considered to be typically benign dysrhythmia that can be seen in adults as well as in children. In adults, it is defined by a cut-off frequency value from 50 to 120 beats per minute (bpm), but in children, as normal frequency depends on age, is defined by a percentage of normal sinus rhythm frequency (within 10–15%) [2].

In the pediatric population, it is usually noted in patients with congenital cardiac defects and is relatively rare without underlying cardiac diseases. Mostly being asymptomatic, it is often an incidental finding on routine electrocardiograms (ECG) [3]. Majority share of patients do have benign, self-limitating course of this condition.

Nevertheless, the performed literature review pointed out the need to separate children in groups according to age into newborns and older children and according to the daily proportion of AIVR into sporadic and frequent, due to differences in clinical sequence of the disease and the prognosis of the diagnosis.

Recommendations for treatment of this dysrhythmia in children are modest. In 2014, a consensus statement was issued suggesting that if the patient is asymptomatic, has no hemodynamic repercussion, no arrhythmogenic myocardial disease, or existing myocardial dysfunction, there are no indications for active treatment. Literature supporting these recommendations do not mention patients with frequent AIVR [4]. Emphasis is placed on individual patient observation and treatment options. If treatment is needed, it can be performed with medication, most often with beta-blockers, and ablation may be considered if there is no adequate control of arrhythmia with antiarrhythmic drugs [4, 5]. However, throughout the entire review of the literature, a lot of cases were presented in which drug treatment was attempted, but no one reported adequate control of arrhythmia in patients with high daily burden [6–8]. Up to now, catheter ablation as a way of treatment has been only sporadically reported in the existing literature. Hence, we present a case of an 11-year-old patient suffering from AIVR, with a large burden of ventricular extrasystoles, in which a successful radiofrequent (RF) ablation was performed.

## Case presentation

An 11-year-old male had been referred to our center from another hospital for a wider workup of incidentally discovered wide-complex arrhythmia. He had never experienced any possible cardiac etiology symptoms such as palpitations, chest pain, dizziness, or syncope. Other than mild tachycardia, his cardiovascular examination as well as the remainder of his physical examination was normal.

**Fig. 1:**
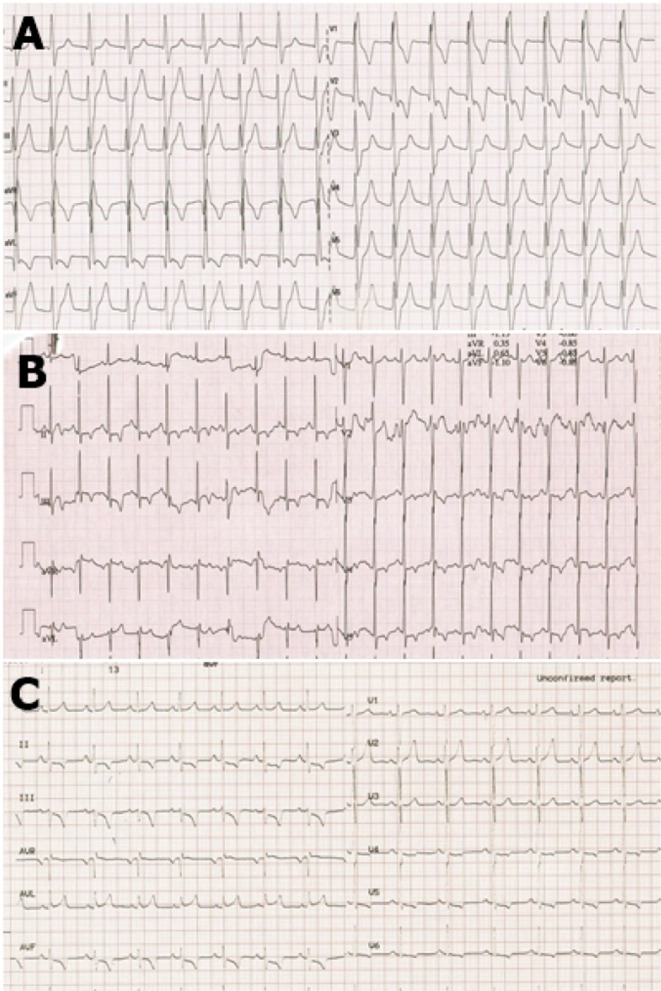
**A** Initial ECG; **B** converting AIVR to sinus rhythm at a threshold heart frequency above 120 bpm during exercise treadmill test; **C** ECG few hours after the procedure

After preforming a 12-lead surface electrocardiogram (ECG), he was placed on a continuous heart rate monitor in-hospital (telemetry) during 4 days where we evidenced accelerated idioventricular rhythm almost constantly during the monitoring, except when in exertion. The QRS morphology of sinus rhythm was normal (QRS width 80 ms; QRS axis intermediate-25°), whereas that of AIVR was right bundle branch block (RBBB) type and superior axis (QRS width 130 ms, QRS axis − 47°). (Fig. 1A).

A 12 lead 24-h Holter-ECG revealed incessant AIVR, consisting up to 73% of the whole record, including isolated monomorphic ventricular extrasystoles (3.5%), with maximum, minimum, and average heart rates of 150 beats per minute (bpm), 49 bpm, and 88 bpm, respectively. During the AIVR, the heart rate was approximately 105 bpm.

The exercise treadmill test revealed converting the idioventricular rhythm to sinus rhythm at a threshold heart frequency of about 120 bpm and higher, as well as returning to the idioventricular rhythm as the frequency got lower during the rest period. (Fig. 1B).

The performed transthoracic echocardiogram showed no underlying structural cardiac disease, with normal contractility and ejection fraction.

During the hospitalization, he remained hemodynamically uncompromised, denying fast or unusual heartbeats.

**Fig. 2:**
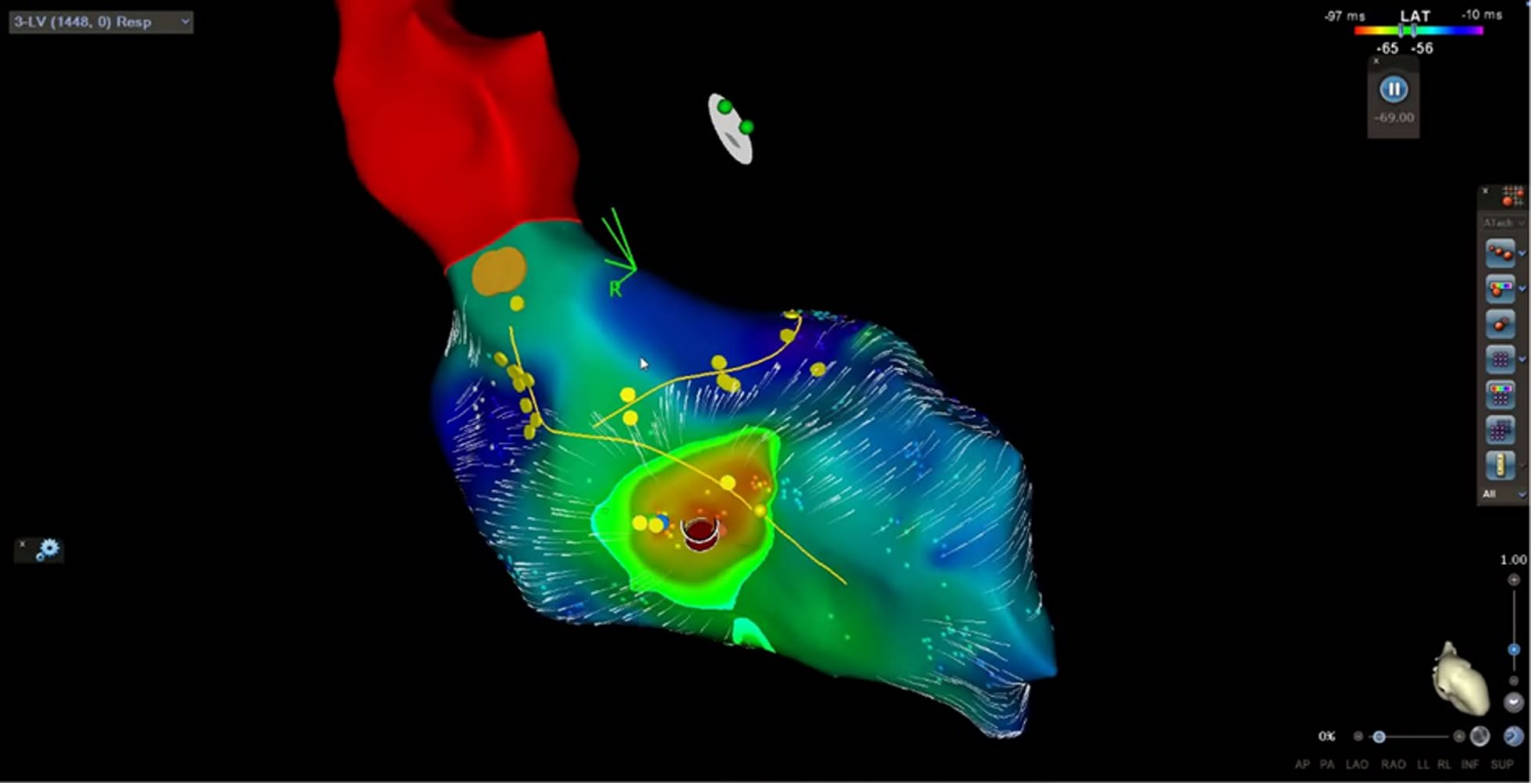
Carto 3D mapping system picture of ectopic focus in the left ventricle, inferior view. Yellow line—His, left branch, anterior and posterior fascicle; dark red dot—the place of mechanical block and successful ablation

After the repeated workup with same findings 2 months later, and with parental consent, we decided to perform an electrophysiological study (EPS). We used the CARTO 3D electroanatomic mapping system (Johnson&Johnson) and placed catheters in the coronary sinus, right ventricle, and the His position. The EP study confirmed the mechanism of tachycardia by entrainment from the right atrium and right ventricle. Afterward, through the femoral artery, the Pentaray catheter was placed in the left ventricle and activation mapping was conducted. His, left branch, anterior and posterior fascicle were marked (Fig. 2). The earliest activation spot was found septal mid-apically (probably papillary muscle area). On this spot, there was no Purkinje signal, which is seen in sinus rhythm. During the mapping, the ventricular rhythm was interrupted, because of transient mechanical block on the earliest activation spot (Fig. 2). Radiofrequent energy was applied (Thermocool ST SF 8F 30 W, power controlled) on mentioned area, after which ventricular rhythm disappeared and normal sinus rhythm appeared. We did four lesions: 60, 40, 30 and 33 s. Target contact force was 10–20 g, and we had 9 do 16 g. Delta impedance was 14 to 19 ohms. Force–time integral was between 436 and 560. Child was constantly in ventricular rhythm, except for short interruption because of mechanical block, so success was evident due to sinus rhythm after less than 10 s of first ablation. Waiting period was 30 min. The procedure was successfully conducted without complications. After conducting catheter ablation, the patient had a normal ECG. (Fig. 1C).

Moreover, during 17 months after the ablation, we conducted a few 24-h Holter-ECG and treadmill tests, all had normal values for the patients age. Table 1 presents a timeline: exhibits the number of ventricular extrasystoles (VES), maximum frequency (F_max_), and average frequency (F_avg_) verified by 24-h Holter-ECGs during evaluation and after the procedure performed in September 2020.

**Table 1:** Timeline-overview of main values verified by 24-h Holter ECG before the procedure (July 2020) and during 17 months follow up

Month, year	VES (n)	F_max_ (bpm)	F_min_ (bpm)
July 2020	95,282	150	88
October 2020	36	181	97
March 2021	0	144	82
May 2022	0	143	72

Bpm, beats per minute; F_avg_, average frequency; F_max_, maximum frequency; VES, ventricular extrasystoles

## Discussion and literature review

AIVR is a wide QRS complex arrhythmia consisting of at least three consecutive, monomorphic ventricular complexes [1]. In children, the normal frequency depends on age and is defined by a percentage of normal sinus rhythm frequency. We consider AIVR to be 10–15% higher than normal, whereas ventricular tachycardia has a rate of more than 15% of normal [9]. Most commonly, it is seen in adults in the reperfusion stage of myocardial infarction [10], whereas in children it is usually related to congenital heart defects, cardiomyopathies, and cardiac tumors. It can be induced by hyperthermia, metabolic or electrolyte dysbalance, myocarditis, or some medications (e.g.digitalis [11], halothane [12], desflurane [13]) [4], or it is isolated, as in this case report. It is thought to be related to enhanced automaticity in His-Purkinje fibers or the working contractile ventricular cells [3]. In our case, we revealed an ectopic focus in posteromedial papillary muscle in a patient without any other underlying condition.

Given the extremely high proportion of wide QRS complexes in form of AIVR in 24-h Holter-ECG as well as on continuous heart monitoring during hospitalization, in this case, we opted for treatment even though the patient was asymptomatic and still did not have evidence of left ventricular dysfunction. We aimed to terminally end this condition and improve patient's future quality of life. Long-term use of antiarrhythmics can potentially have an arrhythmogenic effect, and in AIVR, the success of drug therapy is questionable [6, 7]. Due to the high degree of success of ablation of focal ventricular arrhythmias originating in the papillary muscles in centers of high excellence [12], we opted for the electrophysiologic study by which the source of AIVR was easily detected, and we opted for ablation as a method of treatment, although AIVR has been rarely treated this way heretofore.

A systematic literature search was conducted to identify pediatric patients with AIVR. Please note, that patients with significant systemic illness or with underlying structural or functional heart disease were excluded. MEDLINE /PubMed and Scopus databases were searched (from November 2021 to June 2022).

In total, 82 adequate patients were identified, of whom 44 were newborns and 38 were postneonatal age. These two groups need to be separated in the literature review as well as in clinical treatment due to different clinical courses, outcomes, and prognoses of this dysrhythmia.

With some regularity, AIVR can be seen in otherwise healthy newborns during workup for clinically revealed arrhythmia or is an accidental finding. All described neonatal cases were hemodynamically stable and asymptomatic during arrhythmia, which was transient and self-limiting in otherwise healthy children (Table 2). Attempts of treatment with antiarrhythmic drugs (AAD) have been described but did not prove significant, as the arrhythmia would resolve at different times after drug administration or after discontinuation of pharmacotherapy. The reported children were followed-up from a minimum of 6 to a maximum of 132 months of age and only one of those described had persistent intermittent AIVR at 6 months of age [14].

**Table 2:** Cases of accelerated idioventricular rhythm in neonatal age

Author, year	Patients (n)	Daily AIVR burden (%)	LVEF	Treatment	Follow-up (duration and outcome)
Bergdahl [15]	2	Unknown	Normal	AAD	6 months, 20 months; complete recovery
Bisset [16]	3	Unknown	Normal	AAD 1/3	6 months–2 years; complete recovery
Scagliotti [17]	8	Unknown	Unknown	AAD^•^	unknown
Van Hare [18]	12	Unknown	Unknown	AAD 4/12	2 months–11 years; complete recovery in patients without AAD and in patients on AAD after withdraw; 2 patients died from other causes
MacLellan [6]	2	Unknown	Unknown	No	31 months–16 years; complete recovery
Kurotobi [19]	1	Unknown	Unknown	Unknown	2 years; complete recovery
Anatoliotaki [20]	2	Unknown	Unknown	AAD 1/2	6–18 months; complete recovery
Rehsia [14]	1	“Intermittent”	Normal	AAD	6 months; complete recovery
Roggen [21]	3	Unknown	Unknown	No	unknown*
Freire [22]	4	Unknown	Unknown	AAD 2/4	2 months–1 year; complete recovery
Wang [7]	6	Unknown	Normal	Unknown	Unknown*

AAD, antiarrhythmic drugs; AIVR, accelerated idioventricular rhythm; LVEF, left ventricular ejection fraction. ^•^unknown number of treated; *for these specific patients

In the postneonatal group, 38 children who did not have associated congenital heart disease or other chronic diseases were reported altogether. A larger group of patients was first described by MacLellan-Tobert in her 1995 paper. 12 pediatric patients were described, 2 of whom were newborns and 4 had CHD. The ECG spontaneously normalized in 8. Not one was reported to have symptoms or signs of hemodynamic instability. They were followed up for an average of 68 months [6].

The first review of the literature of the last century was published by Reynolds and Pickoff in 2001 [23], which included 34 children with AIVR, 20 of whom had no associated comorbidities. Less than half had symptoms in terms of palpitations or presyncope. Attempts to treat using AAD were unsuccessful. One patient had “heart enlargement” that was thought to be associated with atrioventricular dissociation and dyssynchrony.

In a vast number of articles published before the 2000s there is no exact data on the duration of arrhythmia, the total daily share of an abnormal rhythm, frequency variability, left ventricular ejection fraction (LVEF), etc.[6, 23], which are today our main backbone of clinical assessment and making decisions regarding follow-up and/or including therapy.

Table 3 shows a simplified overview of recent literature, after Reynolds and Pickoff review in 2001 onwards. The number of published cases from 2001 to date is modest, 18 children in total, most of those being more complicated and complex than previously described which raises the question of publishing the patients with simpler clinical courses and of the true prevalence of AIVR among the healthy pediatric population.

**Table 3:** Cases of accelerated idioventricular rhythm in postneonatal age from 2001 to date

Author, year	Patients (n)	Age (y)	Daily AIVR burden (%)	LVEF (%)	Symptoms	Treatment	Follow-up
Wang [7]	13	1–18	Unknown	“Normal”*	8/13	AAD (6/13)	Unknown•
Chen [24]	1	16	99.6	45	No	RFA	6 months; complete recovery
Chen [24]	1	17	20.9	68	Palpitations	RFA	6 months; complete recovery
Errahmouni [25]	1	12	Unknown	25	Cardiogenic shock	RFA	6 months; complete recovery
Ergul [26]	1	11	90	45	Cardiac arrest	CA	9 months; complete recovery
Kappy [27]	1	13	Unknown	“Normal”	No	No	18 months; complete recovery

AAD, antiarrhythmic drugs; AIVR, accelerated idioventricular rhythm; CA, cryoablation; LVEF, left ventricular ejection fraction; RFA, radiofrequent ablation; *two patients had slightly reduced ejection fraction; •for these specific patients

Wang and co-workers published a paper on ventricular arrhythmias in children in 2010, 77 of them from 1980 to 2010. 19 had AIVR, of whom 6 were newborns. All children were hemodynamically stable, less than half showed symptoms, the most common being palpitations. All underwent 24-h Holter-ECG, and no data on daily burden was available. Cardiac function estimates were normal except slight reduction in ejection fraction in two patients, both spontaneously regressed. 6 were pharmacologically treated which was followed by arrhythmia resolution, but, according to the authors, it is impossible to say whether it was the effect of the drug itself or spontaneous resolution, which occurred in 13 who were not treated. No EPS has been performed [7].

Chen et al. enrolled 8 patients in their study, of whom 2 were boys aged 16 and 17. The 16-year-old was asymptomatic, with a dominant AIVR burden of 99.6%, and LVEF of 45%. 17-year-old had intermittent AIVR, with a daily burden slightly higher than 20%. AAD therapy was tried in both. Complete arrhythmia suppression did not occur. Both underwent an EPS with RF ablation that successfully restored sinus rhythm [24].

In 2017, Errahmouni et al. reported a 12-year-old with previously diagnosed AIVR who acutely presented with heart failure (LVEF 25%) and cardiogenic shock. He was admitted to the intensive care unit (ICU) and treated with mechanical ventilation, electrocardioversion, inotropic drugs, and amiodarone to which the arrhythmia was terminated. Amiodarone therapy was continued after discharge, but when the therapy withdrawal was attempted, the boy was rehospitalized under the same symptoms and treated as before. RF catheter ablation was successfully performed in a steady-state. After 4 weeks, complete recovery of LVEF, with sinus rhythm and RBB in ECG was marked [25].

Ergul et al. reported a case of left coronary cusp cryoablation originating AIVR in an 11-year-old who was previously followed up due to asymptomatic AIVR. He had experienced an aborted cardiac arrest during sleep and had been resuscitated for 5 min. 24-h Holter-ECG revealed incessant AIVR, consisting of up to 90% of the whole record, and two torsades de pointes attacks, triggered by the AIVR-induced “R on T” phenomenon, resulting in syncope and cardiac arrest. Transthoracic echocardiography revealed no structural cardiac defect but mild left ventricular systolic dysfunction. The cryoablation was performed in a steady state. 9 months later, he was asymptomatic, without any PVCs or AIVR, with normal cardiac function [26].

The latest reported case is from 2021 by Kappy et al. It is more similar to the cases described earlier, about an accidental finding of intermittent AIVR in a 13-year-old during post-tonsillectomy bleeding workup. As she stayed asymptomatic and had no cardiac dysfunction, she was discharged without therapy, with a follow-up recommendation in 18 months [27].

As of last, we would like to point out one rare case report of long-term, high-burden AIVR with a consequent decrease in LVEF described by Mine in 2012. A 35-year-old woman had been diagnosed with AIVR for 12 years. It was detected accidentally, the rhythm was persistent with an average sinus-like frequency. At the age of 32, she developed palpitations and shortness of breath on exertion, beta-blocker therapy was introduced, which was ineffective. After three years of therapy, her regular workup showed reduced LVEF, therefore it was decided to perform RFA. After ablation, rhythm was restored to sinus and cardiac function recovered [28]. This is an example of worsening heart function in long-term frequent AIVR. Despite being an adult patient, the question arises- since when did the patient have dysrhythmia? It is a patient who did not have the characteristic features and criteria that would speak of AIVR casuistry corresponding to adulthood as mentioned earlier.

Evidently, in case there is no need for acute treatment due to hemodynamic instability or reduced myocardial function, it is necessary to evaluate the share of wide QRS complexes, in other words, premature ventricular complexes (PVC). This is important since the daily burden of PVCs is the most prominent predictor of cardiomyopathy [29]. One of the rare studies conducted in the pediatric population showed that even a 5% burden of PVCs can cause a decrease in left ventricle function [30]. Baman et al. showed in their study that a cut-off value of 24% of PVCs causes left ventricular dysfunction with about 80% sensitivity and specificity [31]. In PVCs, the burden on the heart function comes not just from the prematurity, but mostly from the dyssynchrony of the contraction [32].

In AIVR, when the frequency is high, heart contractions are mostly in a dyssynchronous manner so we can expect cardiomyopathy somewise comparable to frequent PVCs. Yet there is not enough data on which percentage of PVCs in which period causes impairment in left ventricle function in the case of AIVR. According to Merchant et al., pathophysiologically, cardiomyopathy arises due to impaired, dyssinchron mechanichal myocardium contraction and relaxation, intraventricular as well as interventricular. It occurs in pacemaker-induced cardiomyopathy (PICM), left bundle branch block cardiomyopathy, as well as in premature ventricular contraction-induced cardiomyopathy, which can pathophysiologically be conceptualized as variants of broader dyssynchrony-associated cardiomyopathy. Not only the share but also the width of QRS complexes contributes to dyssynchrony, proportionally [32].

In 2021, Wang et al. published a study they conducted from 2002 to 2018, including 27 patients, in which it was shown that AIVR burden larger than 73.8% could predict impaired left ventricular ejection fraction with 100% sensitivity and 94.1% specificity. Their conclusions go even further, claiming that, unlike transient AIVR, which requires no treatment, frequent AIVR requires optimal treatment, and catheter ablation to be the utter resort in some patients. Also, they find AIVR burden over 70%, impaired LVEF, or syncope/presyncope to be indications for catheter ablation [8].

## Conclusion

The clinical course of AIVR seems to be of low significance in the pediatric population. However, our case added up to not many cases found in the existing literature. The course of this dysrhythmia significantly depends on daily burden of AIVR, which if high, might not be as benign as thought, in meanings of short-term or long-term outcomes.

Unfortunately, with such a low number of similar patients so far, cut-off values ​​or therapeutic direction hadn't been determined, at least not evidence-based. Radiofrequent ablation as a modality of treatment of high burden AIVR in hemodynamically stable patients with normal myocardial function to prevent possible cardiomyopathy is not common.

We believe that reporting various clinical presentations and modalities of therapeutics in AIVR patients is important to achieve improvement of diagnosing, treatment guidelines, and overall understanding of this dysrhythmia.

## Data Availability

All data generated or analysed during this study are included in this published article and/or are available from the corresponding author on reasonable request.
